# Examining the Relationship Between Past Orientation and US Suicide Rates: An Analysis Using Big Data-Driven Google Search Queries

**DOI:** 10.2196/jmir.4981

**Published:** 2016-02-11

**Authors:** Donghyun Lee, Hojun Lee, Munkee Choi

**Affiliations:** ^1^ Korea Advanced Institute of Science and Technology Graduate School of Innovation and Technology Management Daejeon Republic Of Korea; ^2^ Dae-Dong Hospital Department of Pediatrics Busan Republic Of Korea

**Keywords:** attitude, big data, Google search query, Internet search, past orientation, suicide

## Abstract

**Background:**

Internet search query data reflect the attitudes of the users, using which we can measure the past orientation to commit suicide. Examinations of past orientation often highlight certain predispositions of attitude, many of which can be suicide risk factors.

**Objective:**

To investigate the relationship between past orientation and suicide rate by examining Google search queries.

**Methods:**

We measured the past orientation using Google search query data by comparing the search volumes of the past year and those of the future year, across the 50 US states and the District of Columbia during the period from 2004 to 2012. We constructed a panel dataset with independent variables as control variables; we then undertook an analysis using multiple ordinary least squares regression and methods that leverage the Akaike information criterion and the Bayesian information criterion.

**Results:**

It was found that past orientation had a positive relationship with the suicide rate (*P*≤.001) and that it improves the goodness-of-fit of the model regarding the suicide rate. Unemployment rate (*P*≤.001 in Models 3 and 4), Gini coefficient (*P*≤.001), and population growth rate (*P*≤.001) had a positive relationship with the suicide rate, whereas the gross state product (*P*≤.001) showed a negative relationship with the suicide rate.

**Conclusions:**

We empirically identified the positive relationship between the suicide rate and past orientation, which was measured by big data-driven Google search query.

##  Introduction

Recently, the new approach of using big data to find complex or hidden social phenomenon has been trending in different fields of research. In particular, as the Internet is an integral part of the current society, Internet search query data are now considered useful in analyzing consumer behavior [1,2] and disease surveillance [3-5]. In line with this, several studies have examined the relationship between suicide rates and Internet search queries [6-10]. Gunn and Lester [6] found that there is a correlation between search query volume and suicide, using search terms such as “how to suicide” and a cross-sectional study of a US state in 2009. Through time-series data analysis of Japanese data, Hagihara et al [7] found that the number of suicide-related queries tends to increase before the increase in suicide rate. In summary, previous studies have mainly examined the correlation between the suicide rate and Internet search behavior as a warning sign of suicide by examining time-series or cross-sectional data.

Furthermore, Internet search queries and online social media reflect the collective consciousness of the society [1,11,12]. Google’s page rank algorithm is also based on collective consciousness [13]. Preis et al [14] developed the future orientation index of the people of a country by utilizing search query data and calculating the ratio of the future year phrase of the Google search queries index to that of the past year. Through this process, the authors tried to measure a regional preference of future time perspective by quantifying future orientation index. Although we used the orientation index term followed by Preis et al [14] along with time perspective, its mean is the same as the one in this research. This future orientation index has been found to have a positive correlation with the gross domestic product (GDP) of a country [14]. Thus, it is possible to detect the specific attitudes of the populace by examining Internet search query data.

Further, although attitudes are significant reflectors of suicidal tendency among adolescents [15,16], it is difficult to quantify the suicide risk, given the lack of criteria linking personality and suicide [17]. Yufit and colleagues [17,18] insist that distortions of time perspective cause people to commit suicide. Keough et al [19] insist that people categorized their perceptions, events, or plans into the past, present, and future perspectives, and the time perspective was utilized to form memories or goals; thus, it is also possible to predict some behavior such as smoking and drinking by investigating the time perspective. In particular, past orientation was found to create unattained goals [20], and a severe past orientation often related to life regrets [21]. Many depressed persons are troubled by past events and memories, relative to those from the present or aspirations of the future [22]. For these reasons, having a past orientation can be a risk factor for suicide.

However, to the best of our knowledge, no study has considered the regional attitudes—especially the past orientation of residents—and relationship between suicide rate and past orientation using Internet search query. Survey-based measurements of past orientation have limitations. First, it is difficult to collect big-data samples that measure past orientation across various regions over a long-term period, because questionnaire investigations that measure time perspectives tend to be economically infeasible. Second, survey-based measurement can also succumb to social desirability bias. To overcome these limitations, we measured past orientation using Google search query rather than survey data. To be precise, we measured past orientation through the use of modified Preis et al’s [14] future orientation index. These data are derived from the Google portal’s big search query data, and hence, these are reliable. Further, the nature of the data makes it possible to measure past orientation in various countries or regions and over long-term periods, and thus, to construct panel data. Ultimately, we conducted a study under the assumption that past orientation has a positive relationship with the suicide rate; to this end, we examined the relationship between past orientation and suicide rate among the US states between 2004 and 2012 by using panel ordinary least squares (OLS) regression.

More specifically, we measured the past orientation of residents of the United States annually by state, based on big data-driven Google search queries. Next, we controlled independent variables such as the unemployment rate and Gini coefficient. In addition, we arranged the given data as panel data to improve their reliability. Previous suicide studies that utilized Google search queries mainly used time-series and cross-sectional data. Finally, we examined the relationship between suicide rates and past orientation. Besides, to verify robustness of the past orientation variable and our regression model, we calculated the goodness-of-fit of all possible variable combinations through the Akaike information criterion (AIC) and the Bayesian information criterion (BIC) methodologies.

The remainder of this paper is organized as follows. The “Methods” section outlines the methods and variables used to measure past orientation using Google search queries. This section also presents the research model that we use. In the “Results” section, we present the empirical results regarding past orientation and other independent variables by US state. Finally, in the “Discussion” section, we discuss our results, the implications with respect to past orientation, and this study’s limitations.

##  Methods

### Past Orientation, Examined Through Google Search Queries

Wohlford [23] measured the time perspective by examining responses to the Thematic Apperception Test (TAT). In concrete terms, when measuring the time perspective using this test, the participant writes a story that features past, present, or future viewpoints, in accordance with the TAT cards provided. Then, based on the outcome of that story, a score is generated that reflects the participant’s degree of preference for the past versus the future [24]. Furthermore, the Zimbardo Time Perspective Inventory (ZTPI), a questionnaire method to improve subjectivity of TAT, was developed to measure time perspective [19]. ZTPI measures the individual’s time perspective (future, present, and past) through a questionnaire. In summary, the methods of measuring the time perspective center on finding one’s preference vis-à-vis time orientation among the past, present, and future through the questionnaire method.

Similarly, Preis et al [14] quantified the future orientation index of a country by utilizing search query data. To measure the future orientation index, Preis et al [14] calculated the ratio of the future year (eg, ‘‘2010” in 2009) phrase of the Google search queries index to that of the past year (eg, ‘’2008’’ in 2009). We measured the past orientation of state residents each year and calculated the ratio of the past year (eg, ‘‘2008’’ in 2009) phrase of the Google search queries index to that of the future year (eg, ‘‘2010’’ in 2009) by US state. For each year, “past year” and “future year” phrases were changed according to the base year. For example, past orientation in 2010 is the ratio of the “2009” phrase of the Google search queries index to the “2011” phrase of the Google search queries index. For another example, past orientation in 2006 is the ratio of the “2005” phrase of the Google search queries index to the “2007” phrase of the Google search queries index. In this way, we calculated the past orientation of residents of the 50 US states and the District of Columbia between 2004 and 2012. While Preis et al [14] conducted an international comparison of 45 countries for 3 years, we performed an intranational analysis of 50 US states and the District of Columbia for 9 years. As a result, although we controlled variables such as gross state product (GSP), other factors that can affect suicide rate (eg, cultural difference) also needed to be controlled. In addition, if we analyze international countries, aggregating different sources of suicide rate data in different countries is unavoidable; however, this can cause a data quality problem. Based on this criterion, we were able to measure past orientation index by using Google search query data and comparing yearly search volumes in past and future years among residents of the 50 US states and the District of Columbia. In addition, although Preis et al [14] excluded countries with population less than 5 million, because of the possible inaccessibility of search query data due to the low number of search queries, search query data in US states are sufficiently accessible without any exception. Also, in the US, Internet penetration and Google market share are sufficiently high to utilize search query data. According to internetlivestats, penetration rates of Internet in the US in 2004 and 2012 are 64.76% and 81.03%, respectively.

Returning to the model, Equation (1) is the formula we used to quantify past orientation. The method for calculating the Google search query index for each numerator and denominator is identical to that with regard to Google Trends.

Past orientation_*it*_ = 100 × {[Number of Google search – queries for “past year”]_*it*_/[Number of Google search – queries for “past year”]_max_}/{[Number of Google search – queries for “future year”]_*it*_/[Number of Google search – queries for “future year”]_max_} (1)

The numerator in Equation (1), that is, is the index of Google search queries in the past year for state *i* during the year *t*. Specifically, [Number of Google search queries for “Past year”]_max_ is the “past year” search volume that is the largest “past year” search volume among the 50 US states and the District of Columbia in the year *t*. We then calculated the standardized relative proportion of search volume for the “past year” phrase by state *i*.

**Figure 1:**
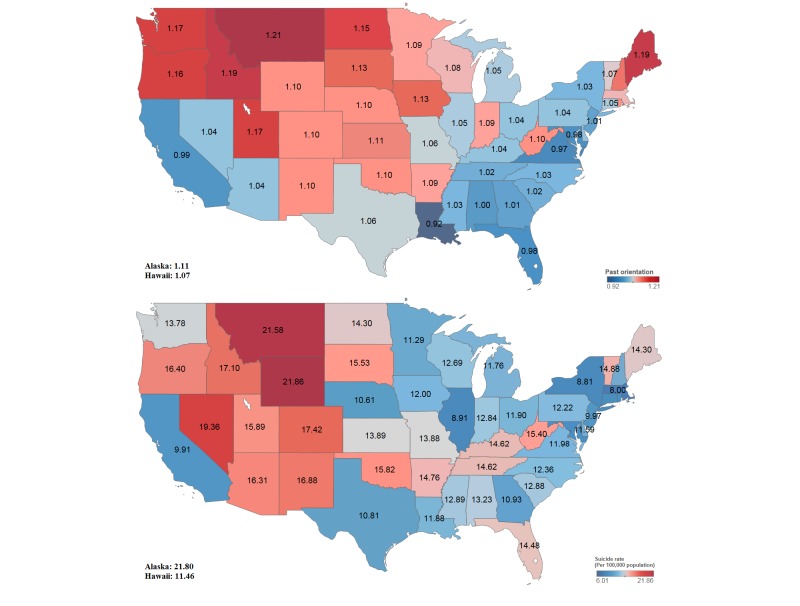
Past orientation index and suicide rates by US state.

Furthermore, the denominator is a Google search query index for “future year” for state *i* during the year *t*. The rest of the calculation is identical to that of the previous denominator for “*past year*.” In more concrete terms, we utilized Google search query data from Google Trends, which provides Google search query data over time and by region, such as by country or state. Finally, the measured past orientation was found to vary from 0.775 to 1.517 by state during the analysis period from 2004 to 2012. Figure 1 shows state-specific average past orientation and average suicide rates.

In Figure 1, Montana, Maine, and Oregon showed a high past orientation value. By contrast, Maryland, California, and Georgia showed a relatively low past orientation value. We examined the past orientation differences among states to determine how they may affect state-specific suicide rates.

### Dependent Variable

Suicide rates for each of the 50 US states and the District of Columbia for the 2004-2012 period were obtained from the Centers for Disease Control and Prevention’s deaths data. The suicide rate unit is the number of suicides per 100,000 population. These data are originally recorded on death certificates and filed on states registration offices, and the suicide statistic is processed by Vital Statistics Cooperative Program of Centers for Disease Control and Prevention [25].

There was some variation among the suicide rates of the US states. Figure 1 shows state-specific average suicide rates. Wyoming, Montana, Nevada, Oregon, and Maine recorded higher average suicide rates; in particular, Montana, Maine, and Oregon had high past orientation values. By contrast, the average recorded suicide rates of Massachusetts, New York, and California were relatively low.

### Independent Variables

In this study, we used independent variables that were mainly used in previous studies. These variables also served as control variables in pinpointing the determinants of suicide in the US states. Detailed descriptions of the variables are presented in Table 1.

#### Gini Coefficient

This variable is an index that indicates the relationship between the population distribution and the distribution obtained, where an index value of 0 signifies complete equality and 1 signifies complete inequality. Gunnell et al [27] used the Gini coefficient variable as an income inequality factor to investigate the determinants of the suicide rate; they found that the Gini coefficient has a statistically significant and positive correlation with the suicide rate. In this study, the Gini coefficient variable was used as a control variable to represent income inequality.

#### Unemployment Rate

The unemployment rate is taken on an annual basis. Yang [28] analyzed the US suicide rate in the 1940-1984 period by using single-equation regression. In that study, the unemployment rate was found to have a significantly positive correlation with the suicide rate of white men. Neumayer [29] also found that the unemployment rate had a positive correlation with the suicide rate. While we used this as a control variable, we also expected it to affect the suicide rate positively.

**Table 1:** Variable descriptions.

Variable	State	Time	Description	Unit	Scale	Source
Suicide rate	O^a^	O	Suicide rate	Per 100,000 population		Centers for Disease Control and Prevention’s deaths data
Unemployment rate	O	O	Unemployment rate	Percentage		Bureau of Labor Statistics: local area unemployment statistics
Gini coefficient	O	O	Gini coefficient, an index showing the relationship between the population distribution and the distribution obtained; 0 = completely equal, 1 = completely unequal conditions	Index (0-1)		US state-level income inequality data [26]
GSP	O	O	Gross state production	USD	/10^6^	US Department of Commerce/Bureau of Economic Analysis/Regional Income Division
Population growth rate	O	O	Population growth rate	Percentage		US Department of Commerce/Bureau of Economic Analysis/Regional Income Division
Past orientation	O	O	A ratio of “past year” phrase of Google search queries index to “future year” phrase of Google search queries index	Index		Google Trends

^a^O: State specific and/or time specific.

#### Gross State Product

The GSP refers to the economic outcome of a state. This variable indicates the degree of wealth by state. Similarly, some studies found income or GDP to have a positive correlation with the suicide rate [30-32], whereas others found a negative correlation [29,33-36]. Specifically, Neumayer [29] examined the relationship between GDP per capita and the suicide rate in 68 countries over the 1980-1999 period. In that study, the GDP per capita was found to have a negative correlation with the suicide rate. By contrast, Hintikka et al [32] found that in Finland, the suicide rate increased whenever the economy was on an upswing, and decreased whenever there was an economic recession. We used GSP as a control variable.

#### Population Growth Rate

A state’s population growth rate is its annual rate of population change. Zhang [37] found that among 60 countries in the 1980-1986 period, the population growth rate negatively correlated with the suicide rate. Durkheim [38] asserted that suicide started with modernization, and Zhang [37] interpreted population growth rate as a modernization indicator. In the past, modernized countries were inclined to have a low population growth rate. However, our study examined an already sufficiently modernized period and place and investigated intranational (US states) comparisons, so it is difficult for this study to represent that state’s population growth rate to indicate the modernization degree. However, because US states with high population growth rate tend to have high number of immigrants or temporal migrants for employment [39], it can negatively affect suicide rate.

#### Past Orientation

This variable consists of Google search query values, as described in the “Methods” section. It is calculated as a ratio of the “past year” phrase of a Google search query index value to the “future year” phrase. We conducted this study under the assumption that past orientation has a positive relationship with the suicide rate. Tables 2 and 3 present the summary statistics (including variance inflation factor for multicollinearity check) and correlation matrix of the data, respectively.

**Table 2:** Summary statistics.

Variable	Number of Observation	Mean	Standard error	Minimum	Maximum	Variance inflation factor
Suicide rate	459	13.350	3.779	4.800	29.700	—
Unemployment rate	459	6.309	2.285	2.500	13.800	1.040
Gini coefficient	459	0.607	0.035	0.536	0.760	1.120
GSP	459	0.281	0.341	0.023	2.100	1.160
Population growth rate	459	0.899	0.887	−5.720	5.290	1.030
Past orientation	459	1.071	0.102	0.775	1.517	1.070

**Table 3:** Correlation matrix (N=459).^a^

	Suicide rate	Unemployment rate	Gini coefficient	GSP	Population growth rate	Past orientation
Suicide rate	1.000					
Unemployment rate	0.100^b^ (.032)	1.000				
Gini coefficient	0.157^c^ (.001)	0.082^d^ (.081)	1.000			
GSP	−0.336^c^ (<.001)	0.134^c^ (.004)	0.296^c^ (<.001)	1.000		
Population growth rate	0.285^c^ (<.001)	−0.128^c^ (.006)	0.087^d^ (.062)	−0.037 (.434)	1.000	
Past orientation	0.281^c^ (<.001)	0.012 (.794)	−0.154^c^ (.001)	−0.238^c^ (<.001)	0.004 (.924)	1.000

^a^ *P* values are provided in parenthesis.

^b^ *P*<.05

^c^ *P*<.01

^d^ *P*<.10

### Monthly Time Variance in Search Queries

**Figure 2:**
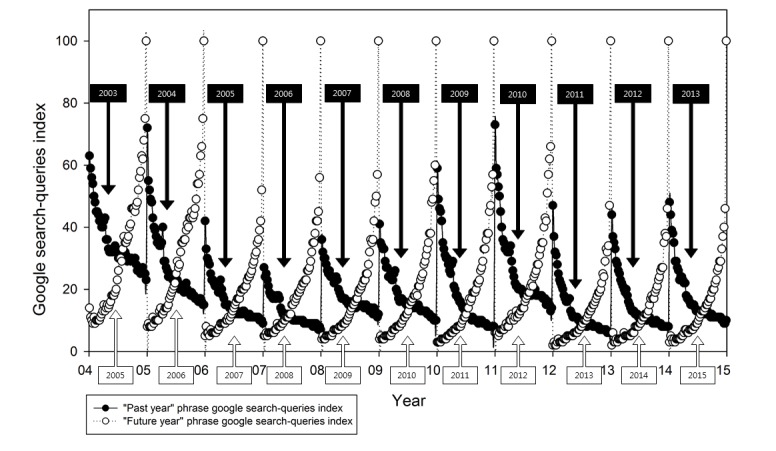
Time variance in Google search queries index values of the past year and future year.

Figure 2 shows the monthly time variance in Google search queries index values of the past year and future year. In Figure 2, the Google search query index values of the past year and future year showed an interesting pattern, depending on the season. The Google search query index for any given past year was highest at the beginning of the year, and it decreased gradually as the year passed. A similar phenomenon was observed in another large-scale big-data study [40]. By contrast, the Google search query index value for the future year showed a gradual increase from the beginning of the year. It then reached its peak when the current year changed to the future year (eg, future search query [“2013”] on December 31, 2012). Additionally, while it can be seen that people gradually change their time perspective focus from the past year to the succeeding year, past-oriented people find it difficult to keep up with a future or present time perspective; indeed, it is not easy for them to depart from the past to which they cling.

### Model

Following many previous studies about suicide rate [28,37,41], we utilized a regression model to investigate the relationship between past orientation and suicide rate. In addition, for using the linear model, we already completed the linearity test through scattering variables. We then calculated the AIC, BIC, and *R* ^2^ for all possible variable combinations for testing the goodness-of-fit and explanatory power of models and variables. Through this process, we determined whether the past orientation variable increases the goodness-of-fit. As such, we were able to investigate whether past orientation is an important variable of the regression model. The following formula is the multiple OLS regression equation that we used:

Suicide rate_*it*_ = α_*t*_ + β_1_ (unemployment rate_*it*_) + β_2_ (Gini coefficient_*it*_) + β_3_ (GSP_*it*_) + β_4_ (population growth rate_*it*_) + β_5_ (past orientation_*it*_) + ∈_*i*_ (2)

where *i* represents a state, *t* the year, and *ϵ*_*i*_ the error term.

We obtained four regression models by combining independent variables. In Model 1, we used only the unemployment rate and the Gini coefficient. In Model 2, we added past orientation to Model 1. We also investigated changes to the AIC and BIC of Models 1 and 2. Through this process, we were able to examine the degree to which the model vis-à-vis past orientation and the suicide rate improved.

The independent variables in Model 3 consist of the unemployment rate, the Gini coefficient, GSP, and population growth rates. Finally, Model 4 added the past orientation variable to Model 3; we also investigated changes to the AIC and BIC in Models 3 and 4.

## Results

### Regression Analysis

Table 4 shows our regression results by model. For both Models 2 and 4, we see that past orientation had a positive relationship with the suicide rate whenever we included the past orientation variable (*P*≤.001). Based on Model 4, when a past orientation value of 1 increases, the suicide rate increases to 8.5 people per 100,000 population (*P*≤.001). These results are consistent with our assumption that past orientation has a positive relationship with the suicide rate. These results indicate that past orientation is often related to life regrets [21] and that it can be a suicide risk factor. In addition, we looked at improvement in the model’s goodness-of-fit with respect to past orientation, and found that in Model 1, the AIC and BIC are 2513 and 2525, respectively, whereas in Model 2, these values are 2468 and 2485, respectively. The AIC and BIC in Model 2 are smaller than those in Model 1, indicating that Model 2 has better goodness-of-fit than Model 1. The AIC and BIC in Model 4 are 2355 and 2379, respectively, whereas these in Model 3 are 2385 and 2405, respectively. As is the case for Models 1 and 2, the AIC and BIC in Model 4 are lower than those in Model 3. In other words, given our AIC and BIC results, it can be said that the use of the past orientation variable improves the goodness-of-fit.

**Table 4:** Regression results.

Variables	Suicide rate											
	Model 1			Model 2			Model 3			Model 4		
	Coeff.	SE	*P* > *t*	Coeff.	SE	*P* > *t*	Coeff.	SE	*P* > *t*	Coeff.	SE	*P* > *t*
Unemployment rate	0.146^a^	0.076	.057	0.133^a^	0.073	.068	0.284^b^	0.067	<.001	0.265^b^	0.065	<.001
Gini coefficient	16.097^b^	4.966	.001	21.284^b^	4.782	<.001	26.230^b^	4.528	<.001	28.568^b^	4.395	<.001
GSP							−4.67^b^	0.467	<.001	−4.122^b^	0.462	<.001
Population growth rate							1.151^b^	0.173	<.001	1.140^b^	0.167	<.001
Past orientation				11.476^b^	1.642	<.001				8.501^b^	1.481	<.001
Cons	2.663	3.017	.378	−12.691^b^	3.615	<.001	−4.078	2.713	.133	−14.623^b^	3.201	<.001
AIC	2513			2468			2385			2355		
BIC	2525			2485			2405				2379	
*P* > *F*	.001			<.001			<.001			<.001		
*R* ^2^	.032			.126			.274			.324		
Adjusted *R* ^2^	.028			.120			.268			.316		
Number of observations	459			459			459			459		

^a^ *P*<.10

^b^ *P*<.01

Next, we find that the unemployment rate has a statistically significant positive relationship with the suicide rate in Models 3 and 4 (*P*≤.001 in Models 3 and 4). Based on Model 4, when the unemployment rate increases to 1%, the suicide rate increases by 0.265 people per 100,000 population (*P*≤.001).

In addition, the Gini coefficient has a statistically significant and positive relationship with the suicide rate (*P*≤.001). Based on Model 4, when the Gini coefficient increases to 1, the suicide rate in a state would increase by about 28.5 people per 100,000 population (*P*≤.001).

The GSP variable in Model 3 was found to have a statistically significant and negative relationship with the suicide rate (*P*≤.001). A high GSP state tends to have a statistically significantly lower suicide rate. Based on Model 4, when the GSP increases by 1 million dollars, the suicide rate decreases by 4.122 people per 100,000 population (*P*≤.001).

Next, the population growth rate variable was found to have a statistically significant and positive relationship with the suicide rate (*P*≤.001). Based on Model 4, when the population growth rate increases by 1%, the suicide rate increases by 1.14 people per 100,000 population (*P*≤.001).

### Goodness-of-Fit of the Regression Model

Furthermore, we verified the goodness-of-fit and explanation power for all possible variable combinations. Table 5 shows the goodness-of-fit of regression results. Model 4 in Table 4, including past orientation and all independent variables, has the smallest AIC and BIC, and the largest *R* ^2^ and adjusted *R* ^2^. In addition, *R* ^2^ of the model, which only has past orientation, is about .079. Its explanation power ranking is third between the independent variables and it is almost similar to the second ranking explanation power. Thus, past orientation also can be a significant factor in a regression model of suicide rate. Lastly, we verified our regression model again through a stepwise regression test with 1% significance level. To conclude, the result is same as that of the goodness-of-fit test, and hence, Model 4 is the best model, consistently.

**Table 5:** Goodness-of-fit of the regression model.

Unemployment rate	Gini coefficient	GSP	Population growth rate	Past orientation	AIC	BIC	*P* > *F*	*R* ^2^	Adjusted *R* ^2^
O^a^	O	O	O	O	2354.5	2379.3	<.001	.324	.316
	O	O	O	O	2368.9	2389.6	<.001	.299	.293
O	O	O	O		2384.8	2405.4	<.001	.274	.268
O		O	O	O	2393.5	2414.1	<.001	.261	.254
O	O	O		O	2397.5	2418.1	<.001	.254	.248
	O	O	O		2400.4	2416.9	<.001	.246	.241
	O	O		O	2404.8	2421.3	<.001	.239	.234
		O	O	O	2409.6	2426.1	<.001	.231	.226
O		O	O		2415.5	2432.0	<.001	.221	.216
O	O	O			2425.6	2442.1	<.001	.204	.198
O	O		O	O	2426.8	2447.5	<.001	.205	.198
		O	O		2432.6	2445.0	<.001	.188	.184
	O		O	O	2432.8	2449.3	<.001	.191	.186
	O	O			2434.0	2446.4	<.001	.185	.182
O			O	O	2440.4	2457.0	<.001	.177	.172
O		O		O	2441.9	2458.4	<.001	.175	.169
			O	O	2448.4	2460.8	<.001	.159	.156
		O		O	2450.3	2462.7	<.001	.156	.152
O		O			2461.6	2474.0	<.001	.135	.131
O	O			O	2468.1	2484.6	<.001	.126	.120
	O			O	2469.5	2481.9	<.001	.120	.116
		O			2470.9	2479.2	<.001	.113	.111
O	O		O		2474.1	2490.6	<.001	.115	.109
O			O		2479.5	2491.9	<.001	.100	.096
	O		O		2480.2	2492.6	<.001	.099	.095
O				O	2485.7	2498.1	<.001	.088	.084
			O		2487.1	2495.4	<.001	.081	.079
				O	2488.4	2496.6	<.001	.079	.077
O	O				2512.9	2525.3	.001	.032	.028
	O				2514.6	2522.8	.001	.025	.023
O					2521.4	2529.6	.032	.010	.008

^a^O: Variable utilized in the model.

### Past Orientation Versus Suicide Rate

**Figure 3:**
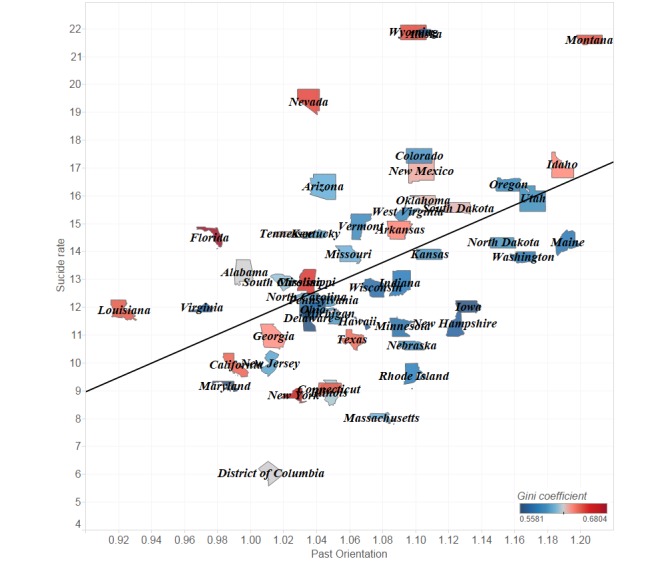
Past orientation versus suicide rate by US state.

Finally, we mapped the average past orientation and the average suicide rate by state (Figure 3). Although there are many factors that affect a state’s suicide rate, we plot between past orientation and suicide rate to focus on past orientation. Notably, the past orientation and suicide rate values vary widely among the US states. As seen in our regression results, there is a tendency where the higher the past orientation of the residents of the state, the higher the state’s suicide rate. In particular, many states with high suicide rates (eg, Oregon, Colorado, and New Mexico) have high past orientation; the past orientation of Georgia, California, and Maryland is low, and their suicide rates are also relatively low. As a result, past orientation—including attachment to the past—can be seen as a suicidal risk factor, and it is found to have a positive correlation with the suicide rate.

##  Discussion

### Overview

We investigated the potential impact of past orientation on suicide rates; we measured past orientation through the use Google search query data. We also found that suicide rates vary widely by state. We built a yearly panel dataset by considering the categorized control variables for the US states in the 2004-2012 period. We were then able to reveal the relationship between past orientation and the suicide rate through multiple OLS regression: past orientation was found to have a positive relationship with suicide rate in a statistically significant manner (*P*≤.001). In addition, through AIC and BIC analyses, past orientation was confirmed as being an important variable of suicide rate in the US states. Ultimately, we were able to pinpoint the relationship between suicide rate and its risk factors in the US states.

### Principal Findings

We have made three salient contributions to the suicide literature. First, we were able to empirically identify the relationship between past orientation and suicide rate. At the individual level, the risk of suicide tends to increase when one faces a divorce or the death of a loved one, is dismissed from work, or experiences health problems, inter alia. These situations are worse than the aforementioned situations (eg, economic status, job stability, and health status) and are specific to a situation where one has lost a relationship with the people around him or her. When one falls into such a situation, he/she tends to focus on the past and may fall into obsession. These also can be one of reasons why past orientation has a positive relationship with the suicide rates. Second, we were able to measure the past orientation of the residents of US states by applying big data-driven Google search query to the phenomenon of suicide. Finally, we were able to verify clearly that the unemployment rate, Gini coefficient, GSP, and population growth rate are the determinants of the suicide rate in the United States.

More specifically, the unemployment rate finding accords with our expectation that it affects the suicide rate positively. This is consistent with the results of previous studies—such as those of Yang [28] and Neumayer [29], who examined national suicide rate determinants. In particular, vulnerable social groups face unemployment and life hardships whenever the unemployment rate is high; therefore, the unemployment rate has a positive relationship with the suicide rate.

In addition, Gini coefficient was found to have a statistically significant and positive relationship with the suicide rate. This result is consistent with the findings of Gunnell et al [27] in England and Wales. This can be interpreted as follows: deepening wealth inequality has a positive association with the high suicide rate. As a result, not only the GSP but also wealth inequality is an important factor of suicide rate.

By contrast, GSP was found to have a negative relationship vis-à-vis the suicide rate. There are strong links between GSP and suicide rate. While income or GDP variables have been frequently considered in many previous studies [29-36], the results thereof have not been consistent. Our results align with those of previous studies that found GSP to have a negative correlation with the suicide rate [29,33-36]. However, they are inconsistent with some studies that found income or economic boom to have a positive correlation with the suicide rate [30-32]. Although it is possible to explain modernization factor as the reason for income or economic factors affecting the suicide rate in these studies [30-32], our study investigated an already sufficiently modernized region and period, and hence, the results may differ.

Finally, the result of population growth rate can be interpreted as follows: states with a high population growth rate can be more changeable and unstable because immigrants and temporal job opportunities are critical reason of population growth [39]. This instability could contribute to a high suicide rate.

### Limitations

Although this study considered many aspects, it nonetheless has some limitations. First, Google search queries data are accessible only from 2004. In addition, the initial stage of Google search queries data, such as data in 2004, is of relatively low reliability because of relatively low Internet users (penetration rate of Internet in the United States is about 64.76% in 2004, but 81.03% in 2012).

In addition, although Google trends provide data only when they have sufficient search query data, a low sample error may occur because of the relatively small population in some states such as Wyoming or Vermont. Finally, Google search query data reflect only the views of people who can access the Internet and Google; for this reason, we cannot reflect on people with no access to the Internet.

### Future Research

Future studies need to investigate the causal relationship between past orientation and suicide rate. This can boost our results and bridge the gap of interventions directed at influencing behavior and attitude. Furthermore, next studies will be conducted on the development of tools by which Internet users can request medical help; on the basis of these findings, such tools would leverage past orientation. It can also be valuable to examine how the government can effectively intervene in suicide risk situations using big-data analysis. We will also try to investigate the relationship between suicide or disorders and other specific attitudes by undertaking big-data analysis made possible by the provision of search query or social network data.

## Abbreviations

AIC: Akaike information criterion
BIC: Bayesian information criterion
GDP: gross domestic product
GSP: Gross state product
OLS: Ordinary least squares
TAT: Thematic Apperception Test
